# Bis(2,6-dihy­droxy­benzoato-κ^2^ *O* ^1^ *,O* ^1′^)(nitrato-κ^2^ *O*,*O*′)bis­(1,10-phenanthroline-κ^2^ *N*,*N*′)gadolinium(III)

**DOI:** 10.1107/S1600536810047124

**Published:** 2010-11-20

**Authors:** Junjia Zheng, Hongxiao Jin, Hongliang Ge

**Affiliations:** a College of Materials Science and Engineering, China Jiliang University, Hangzhou 310018, People’s Republic of China

## Abstract

In the mononuclear title complex, [Gd(C_7_H_5_O_3_)_2_(NO_3_)(C_12_H_8_N_2_)_2_], the Gd atom is in a pseudo-bicapped square-anti­prismatic geometry formed by four N atoms from two chelating 1,10-phenanthroline (phen) ligands and by six O atoms, four from two 2,6-dihy­droxy­benzoate (DHB) ligands and the other two from a nitrate anion. π–π stacking inter­actions between phen–DHB [centroid–centroid distances = 3.5334 (18) and 3.8414 (16) Å] and phen–phen [face-to-face separation = 3.4307 (17) Å] ligands of adjacent complex molecules stabilize the crystal structure. Intra­molecular O—H⋯O hydrogen bonds are observed in the DHB ligands.

## Related literature

For background to the complexation of Gd(III) ions with multidentate ligands with *O*- and *N*-donors, see: Kido & Okamoto (2002 ▶); Lauffer (1990 ▶). For related structures, see: Ma *et al.* (2010 ▶); Wang *et al.* (2008 ▶); Xia *et al.* (2007 ▶).
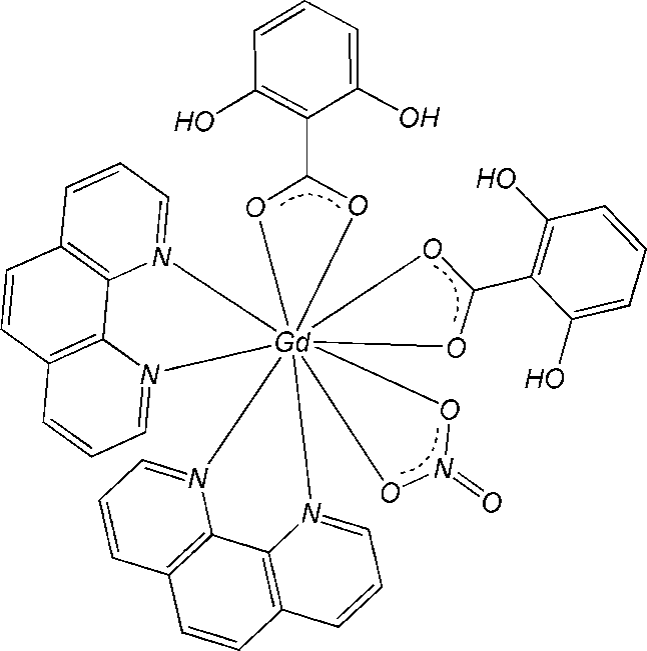


## Experimental

### 

#### Crystal data

[Gd(C_7_H_5_O_3_)_2_(NO_3_)(C_12_H_8_N_2_)_2_]
*M* *_r_* = 885.89
Monoclinic, 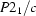

*a* = 11.1623 (2) Å
*b* = 26.7666 (4) Å
*c* = 14.2979 (4) Å
β = 127.445 (1)°
*V* = 3391.60 (12) Å^3^
*Z* = 4
Mo *K*α radiation
μ = 2.03 mm^−1^
*T* = 298 K
0.46 × 0.42 × 0.40 mm

#### Data collection

Oxford Diffraction Gemini S Ultra diffractometer
Absorption correction: multi-scan [*ABSPACK* in *CrysAlis PRO RED* (Oxford Diffraction, 2006 ▶)] *T*_min_ = 0.455, *T*_max_ = 0.497
18394 measured reflections
5979 independent reflections
4815 reflections with *I* > 2σ(*I*)
*R*_int_ = 0.024

#### Refinement

*R*[*F* ^2^ > 2σ(*F* ^2^)] = 0.021
*wR*(*F* ^2^) = 0.043
*S* = 1.01
5979 reflections
496 parameters
12 restraints
H-atom parameters constrained
Δρ_max_ = 0.46 e Å^−3^
Δρ_min_ = −0.47 e Å^−3^

### 

Data collection: *CrysAlis PRO CCD* (Oxford Diffraction, 2006 ▶); cell refinement: *CrysAlis PRO CCD*; data reduction: *CrysAlis PRO RED* (Oxford Diffraction, 2006 ▶); program(s) used to solve structure: *SHELXS97* (Sheldrick, 2008 ▶); program(s) used to refine structure: *SHELXL97* (Sheldrick, 2008 ▶); molecular graphics: *DIAMOND* (Brandenburg & Berndt, 1999 ▶); software used to prepare material for publication: *SHELXL97*.

## Supplementary Material

Crystal structure: contains datablocks I, global. DOI: 10.1107/S1600536810047124/pk2280sup1.cif

Structure factors: contains datablocks I. DOI: 10.1107/S1600536810047124/pk2280Isup2.hkl

Additional supplementary materials:  crystallographic information; 3D view; checkCIF report

## Figures and Tables

**Table 1:** Hydrogen-bond geometry (Å, °)

*D*—H⋯*A*	*D*—H	H⋯*A*	*D*⋯*A*	*D*—H⋯*A*
O7—H7⋯O5	0.82	1.87	2.592 (2)	147
O8—H8⋯O6	0.82	1.83	2.563 (3)	148
O4—H4⋯O1	0.82	1.86	2.585 (3)	147
O3—H3⋯O2	0.82	1.84	2.574 (3)	148

**Fig. 1.**
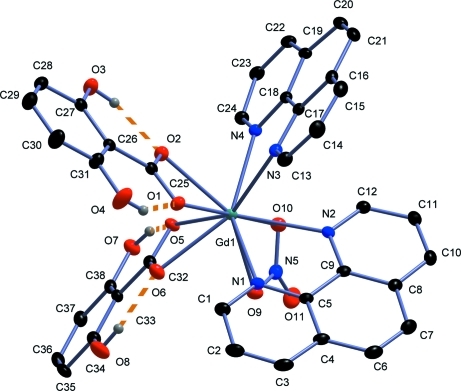
The structure, showing the atom-labelling scheme. Displacement ellipsoids are drawn at the 15% probablility level. Some H atoms are omitted for clarity. Dashed lines indicate the intramolecular O–H···O hydrogen bonds.

**Fig. 2.**
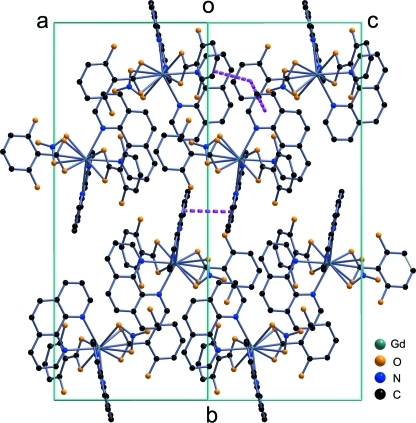
A packing diagram of the unit cell. Pink dashed lines show π–π stacking between ligands.

## supplementary crystallographic information

### Comment

The chemistry of lanthanide-based metal-organic frameworks is currently of great interest because of their unusual coordination characteristics and optical and magnetic properties. The complexation of gadolinium (III) ions with multidentate ligands with O– and N-donors, has received great attention because of its relevance in biomedical applications as magnetic resonance imaging (MRI) agents (Kido & Okamoto, 2002; Lauffer, 1990). Herein, we report on the preparation and the single-crystal X-ray structure behavior of the novel mononuclear mixed-ligand complex [Gd(C_12_H_8_N_2_)_2_ (C_7_H_8_O_3_)_2_(NO_3_)].

The mononuclear structure is shown in Fig. 1. The ten-coordinate geometry of the Gd^III^ ion is completed by four 2, 6-dihydroxybenzoate (DHB) O atoms, four phenanthroline N atoms and two nitrate O atoms. In one unit cell, there are four complex molecules (Fig. 2).

The complex forms a ten-coordinate pseudo-bicapped square antiprismatic structure in which the set of O5, O9, N2 and N4 and the set of O2, O6, N1 and N3 form two approximate squares, respectively. The ninth coordinate atom O1 and tenth coordinate atom O10 are above and under the two planes formed by O5, O9, N2 and N4 and of O2, O6, N1 and N3, respectively, and locate at bicapped positions. The O2–Gd–O9 is 175.388 (1)°, close to 180°. Because the coordinate O1 and O10 atoms are excluded by O5, O9, N2 and N4 (forming the above plane) and O2, O6, N1 and N3 (forming the plane beneath), respectively, the bond distances of Gd^III^–O1 (2.5673 (16) Å) and Gd^III^–O10 (2.6022 (17) Å) are correspondingly longer than those of Gd^III^–O2 (2.4781 (17) Å) and Gd^III^–O9 (2.5039 (17) Å), respectively.

π··· π stacking is observed in the crystal structure (Fig. 2). The centroid-centroid distances between the phen and DHB are 3.5334 (18) and 3.8414 (16) Å, while the face-to-face separation between parallel phen ligands is 3.4307 (17) Å.

Intramolecular O–H···O hydrogen bonds are observed in the DHB ligands (Fig. 1 & Table 1).

### Experimental

Each reagent was commercially available and of analytical grade. GdNO_3_^.^6H_2_O (0.226 g, 0.5 mmol), 2, 6-dihydroxybenzoic acid (0.074 g 0.5 mmol), 1, 10-phenanthroline (0.090 g, 0.5 mmol) and NaHCO_3_ (0.042 g, 0.5 mmol) were dissolved in water-ethanol solution (10 ml, 5:5). The solution was refluxed for 4 h, and filtered after cooling to room temperature. Orange single crystals were obtained from the filtrate after 1 d.

### Refinement

H atoms were positioned geometrically (C—H = 0.93 Å and O—H = 0.82 Å) and refined as riding, with *U*_iso _(H) = 1.2Ueq (C) and *U*_iso_(H) = 1.5Ueq (O). The displacement parameters of N atom in nitrate and one O atom in DHB were restrained to be equal, while one O atom and N atom in nitrate were restrained to be approximately isotropic.

### Crystal data

**Table:**

[Gd(C_7_H_5_O_3_)_2_(NO_3_)(C_12_H_8_N_2_)_2_]	*F*(000) = 1764
*M**_r_* = 885.89	*D*_x_ = 1.735 Mg m^−^^3^
Monoclinic, *P*2_1_/*c*	Mo *K*α radiation, λ = 0.71073 Å
Hall symbol: -P 2ybc	Cell parameters from 11955 reflections
*a* = 11.1623 (2) Å	θ = 2.9–29.1°
*b* = 26.7666 (4) Å	µ = 2.03 mm^−^^1^
*c* = 14.2979 (4) Å	*T* = 298 K
β = 127.445 (1)°	Block, orange
*V* = 3391.60 (12) Å^3^	0.46 × 0.42 × 0.40 mm
*Z* = 4	

### Data collection

**Table:**

Oxford Diffraction Gemini S Ultra diffractometer	5979 independent reflections
Radiation source: Enhance (Mo) X-ray Source	4815 reflections with *I* > 2σ(*I*)
graphite	*R*_int_ = 0.024
Detector resolution: 15.9 pixels mm^-1^	θ_max_ = 25.0°, θ_min_ = 2.9°
ω scans	*h* = −13→13
Absorption correction: multi-scan (*CrysAlis PRO*; Oxford Diffraction, 2006)	*k* = −31→31
*T*_min_ = 0.455, *T*_max_ = 0.497	*l* = −17→15
18394 measured reflections	

### Refinement

**Table:**

Refinement on *F*^2^	Primary atom site location: structure-invariant direct methods
Least-squares matrix: full	Secondary atom site location: difference Fourier map
*R*[*F*^2^ > 2σ(*F*^2^)] = 0.021	Hydrogen site location: inferred from neighbouring sites
*wR*(*F*^2^) = 0.043	H-atom parameters constrained
*S* = 1.01	*w* = 1/[σ^2^(*F*_o_^2^) + (0.020*P*)^2^] where *P* = (*F*_o_^2^ + 2*F*_c_^2^)/3
5979 reflections	(Δ/σ)_max_ = 0.001
496 parameters	Δρ_max_ = 0.46 e Å^−^^3^
12 restraints	Δρ_min_ = −0.47 e Å^−^^3^

### Special details

**Table:**

Geometry. All e.s.d.'s (except the e.s.d. in the dihedral angle between two l.s. planes) are estimated using the full covariance matrix. The cell e.s.d.'s are taken into account individually in the estimation of e.s.d.'s in distances, angles and torsion angles; correlations between e.s.d.'s in cell parameters are only used when they are defined by crystal symmetry. An approximate (isotropic) treatment of cell e.s.d.'s is used for estimating e.s.d.'s involving l.s. planes.
Refinement. Refinement of *F*^2^ against ALL reflections. The weighted *R*-factor *wR* and goodness of fit *S* are based on *F*^2^, conventional *R*-factors *R* are based on *F*, with *F* set to zero for negative *F*^2^. The threshold expression of *F*^2^ > σ(*F*^2^) is used only for calculating *R*-factors(gt) *etc*. and is not relevant to the choice of reflections for refinement. *R*-factors based on *F*^2^ are statistically about twice as large as those based on *F*, and *R*- factors based on ALL data will be even larger.

### Fractional atomic coordinates and isotropic or equivalent isotropic displacement parameters (Å^2^)

**Table:**

	*x*	*y*	*z*	*U*_iso_*/*U*_eq_	
Gd1	0.930008 (14)	0.862284 (4)	0.221059 (11)	0.03241 (5)	
O1	0.8890 (2)	0.91866 (6)	0.05856 (15)	0.0451 (5)	
O2	0.8450 (2)	0.83835 (7)	0.02180 (15)	0.0443 (4)	
O3	0.7536 (2)	0.80218 (7)	−0.17849 (17)	0.0606 (5)	
H3	0.7812	0.8019	−0.1106	0.091*	
O4	0.8349 (3)	0.97874 (7)	−0.1046 (2)	0.0816 (7)	
H4	0.8533	0.9705	−0.0419	0.122*	
O5	0.69658 (19)	0.81249 (6)	0.13465 (16)	0.0434 (4)	
O6	0.66669 (19)	0.89321 (6)	0.09915 (17)	0.0505 (5)	
O7	0.4812 (2)	0.75919 (7)	0.10366 (17)	0.0542 (5)	
H7	0.5652	0.7649	0.1220	0.081*	
O8	0.4200 (2)	0.93646 (8)	0.0290 (2)	0.0753 (7)	
H8	0.5060	0.9337	0.0492	0.113*	
O9	0.8459 (2)	0.87228 (7)	0.34724 (17)	0.0506 (5)	
O10	0.9519 (2)	0.80131 (6)	0.37257 (17)	0.0492 (5)	
O11	0.8610 (3)	0.81841 (9)	0.46695 (19)	0.0817 (7)	
N1	0.9754 (2)	0.95435 (7)	0.29187 (19)	0.0368 (5)	
N2	1.1640 (2)	0.88032 (7)	0.43955 (18)	0.0357 (5)	
N3	1.1797 (2)	0.86803 (7)	0.24844 (18)	0.0375 (5)	
N4	1.0554 (2)	0.77981 (7)	0.23851 (18)	0.0355 (5)	
N5	0.8865 (3)	0.82976 (9)	0.3979 (2)	0.0470 (6)	
C1	0.8820 (3)	0.99069 (10)	0.2201 (3)	0.0471 (7)	
H1	0.8063	0.9831	0.1414	0.057*	
C2	0.8935 (4)	1.03981 (10)	0.2589 (3)	0.0534 (8)	
H2	0.8261	1.0641	0.2065	0.064*	
C3	1.0030 (4)	1.05157 (10)	0.3726 (3)	0.0517 (8)	
H3A	1.0116	1.0841	0.3989	0.062*	
C4	1.1037 (3)	1.01476 (9)	0.4510 (3)	0.0407 (7)	
C5	1.0847 (3)	0.96619 (9)	0.4060 (2)	0.0343 (6)	
C6	1.2220 (3)	1.02442 (10)	0.5721 (3)	0.0510 (8)	
H6	1.2346	1.0566	0.6013	0.061*	
C7	1.3153 (3)	0.98818 (11)	0.6447 (3)	0.0530 (8)	
H7A	1.3911	0.9957	0.7234	0.064*	
C8	1.3011 (3)	0.93797 (10)	0.6037 (2)	0.0403 (6)	
C9	1.1850 (3)	0.92708 (9)	0.4844 (2)	0.0347 (6)	
C10	1.3956 (3)	0.89923 (11)	0.6760 (3)	0.0496 (7)	
H10	1.4723	0.9051	0.7554	0.060*	
C11	1.3754 (3)	0.85270 (10)	0.6303 (3)	0.0475 (7)	
H11	1.4387	0.8265	0.6774	0.057*	
C12	1.2586 (3)	0.84516 (10)	0.5121 (2)	0.0421 (7)	
H12	1.2458	0.8132	0.4818	0.050*	
C13	1.2402 (3)	0.91126 (10)	0.2505 (2)	0.0475 (7)	
H13	1.1814	0.9400	0.2262	0.057*	
C14	1.3874 (3)	0.91551 (12)	0.2872 (3)	0.0566 (8)	
H14	1.4255	0.9465	0.2875	0.068*	
C15	1.4749 (3)	0.87395 (12)	0.3226 (3)	0.0565 (8)	
H15	1.5739	0.8765	0.3485	0.068*	
C16	1.4167 (3)	0.82720 (11)	0.3202 (2)	0.0436 (7)	
C17	1.2668 (3)	0.82630 (9)	0.2817 (2)	0.0360 (6)	
C18	1.1987 (3)	0.77906 (9)	0.2718 (2)	0.0350 (6)	
C19	1.2788 (3)	0.73454 (10)	0.2958 (2)	0.0412 (7)	
C20	1.4327 (3)	0.73749 (12)	0.3397 (2)	0.0541 (8)	
H20	1.4884	0.7082	0.3602	0.065*	
C21	1.4990 (3)	0.78140 (12)	0.3522 (2)	0.0542 (8)	
H21	1.5998	0.7821	0.3820	0.065*	
C22	1.2026 (3)	0.68959 (10)	0.2755 (2)	0.0482 (7)	
H22	1.2507	0.6593	0.2871	0.058*	
C23	1.0593 (3)	0.69013 (10)	0.2392 (2)	0.0478 (7)	
H23	1.0075	0.6604	0.2246	0.057*	
C24	0.9899 (3)	0.73592 (10)	0.2237 (2)	0.0435 (7)	
H24	0.8926	0.7358	0.2020	0.052*	
C25	0.8459 (3)	0.88219 (10)	−0.0120 (2)	0.0375 (6)	
C26	0.7966 (3)	0.89003 (9)	−0.1327 (2)	0.0357 (6)	
C27	0.7529 (3)	0.84952 (10)	−0.2109 (2)	0.0420 (7)	
C28	0.7051 (3)	0.85738 (11)	−0.3242 (3)	0.0534 (8)	
H28	0.6756	0.8305	−0.3752	0.064*	
C29	0.7015 (3)	0.90493 (13)	−0.3609 (3)	0.0605 (8)	
H29	0.6690	0.9100	−0.4375	0.073*	
C30	0.7443 (4)	0.94522 (12)	−0.2884 (3)	0.0633 (9)	
H30	0.7408	0.9772	−0.3154	0.076*	
C31	0.7930 (3)	0.93800 (11)	−0.1744 (3)	0.0512 (7)	
C32	0.6141 (3)	0.85113 (10)	0.1004 (2)	0.0422 (7)	
C33	0.4596 (3)	0.84803 (9)	0.0660 (2)	0.0386 (6)	
C34	0.3692 (3)	0.89083 (11)	0.0327 (2)	0.0508 (7)	
C35	0.2258 (3)	0.88797 (13)	0.0027 (3)	0.0600 (8)	
H35	0.1660	0.9164	−0.0201	0.072*	
C36	0.1739 (3)	0.84251 (13)	0.0071 (3)	0.0583 (8)	
H36	0.0775	0.8406	−0.0132	0.070*	
C37	0.2579 (3)	0.79927 (12)	0.0404 (2)	0.0520 (8)	
H37	0.2190	0.7690	0.0427	0.062*	
C38	0.4019 (3)	0.80189 (11)	0.0705 (2)	0.0426 (7)	

### Atomic displacement parameters (Å^2^)

**Table:**

	*U*^11^	*U*^22^	*U*^33^	*U*^12^	*U*^13^	*U*^23^
Gd1	0.03358 (7)	0.02755 (7)	0.03929 (8)	0.00033 (6)	0.02381 (6)	−0.00046 (6)
O1	0.0562 (12)	0.0395 (11)	0.0428 (12)	−0.0024 (9)	0.0317 (10)	−0.0020 (9)
O2	0.0514 (12)	0.0361 (11)	0.0439 (12)	−0.0019 (9)	0.0282 (10)	0.0033 (9)
O3	0.0691 (14)	0.0414 (12)	0.0488 (13)	−0.0016 (10)	0.0242 (11)	−0.0072 (10)
O4	0.141 (2)	0.0415 (13)	0.0582 (15)	−0.0182 (14)	0.0586 (16)	−0.0043 (11)
O5	0.0347 (10)	0.0386 (11)	0.0571 (13)	0.0035 (9)	0.0281 (10)	0.0013 (9)
O6	0.0426 (11)	0.0421 (12)	0.0640 (14)	0.0030 (9)	0.0310 (11)	0.0097 (9)
O7	0.0449 (11)	0.0497 (12)	0.0694 (15)	0.0009 (10)	0.0354 (12)	0.0074 (10)
O8	0.0592 (14)	0.0560 (14)	0.102 (2)	0.0174 (12)	0.0444 (14)	0.0139 (13)
O9	0.0563 (11)	0.0455 (12)	0.0608 (13)	0.0035 (9)	0.0412 (10)	−0.0022 (9)
O10	0.0541 (12)	0.0383 (11)	0.0579 (13)	0.0010 (9)	0.0354 (11)	0.0016 (9)
O11	0.0799 (16)	0.124 (2)	0.0590 (16)	−0.0099 (15)	0.0517 (14)	0.0115 (14)
N1	0.0465 (13)	0.0265 (11)	0.0444 (15)	0.0023 (10)	0.0312 (12)	0.0038 (10)
N2	0.0358 (12)	0.0318 (12)	0.0413 (13)	0.0038 (10)	0.0244 (11)	0.0025 (10)
N3	0.0377 (12)	0.0396 (13)	0.0400 (13)	−0.0057 (10)	0.0261 (11)	−0.0030 (10)
N4	0.0362 (12)	0.0331 (12)	0.0399 (14)	−0.0023 (10)	0.0244 (11)	−0.0025 (10)
N5	0.0413 (12)	0.0588 (15)	0.0441 (14)	−0.0096 (11)	0.0275 (11)	−0.0031 (11)
C1	0.0557 (18)	0.0389 (16)	0.054 (2)	0.0056 (14)	0.0374 (17)	0.0061 (14)
C2	0.074 (2)	0.0281 (16)	0.077 (3)	0.0104 (15)	0.056 (2)	0.0109 (15)
C3	0.075 (2)	0.0262 (15)	0.083 (3)	−0.0060 (15)	0.063 (2)	−0.0072 (16)
C4	0.0507 (17)	0.0308 (15)	0.062 (2)	−0.0094 (13)	0.0457 (17)	−0.0074 (14)
C5	0.0390 (15)	0.0303 (14)	0.0474 (18)	−0.0039 (12)	0.0334 (15)	−0.0031 (12)
C6	0.061 (2)	0.0370 (17)	0.076 (2)	−0.0197 (15)	0.052 (2)	−0.0204 (16)
C7	0.0485 (18)	0.057 (2)	0.059 (2)	−0.0200 (16)	0.0356 (17)	−0.0208 (17)
C8	0.0352 (15)	0.0462 (17)	0.0482 (18)	−0.0081 (13)	0.0299 (15)	−0.0077 (14)
C9	0.0380 (15)	0.0339 (15)	0.0472 (18)	−0.0058 (12)	0.0336 (15)	−0.0044 (12)
C10	0.0332 (15)	0.068 (2)	0.0407 (18)	−0.0031 (14)	0.0189 (14)	−0.0040 (15)
C11	0.0393 (16)	0.055 (2)	0.0460 (19)	0.0062 (14)	0.0246 (15)	0.0073 (14)
C12	0.0449 (16)	0.0393 (16)	0.0453 (19)	0.0035 (13)	0.0291 (16)	0.0007 (13)
C13	0.0537 (18)	0.0454 (17)	0.0510 (19)	−0.0092 (14)	0.0358 (16)	−0.0039 (14)
C14	0.0531 (19)	0.060 (2)	0.061 (2)	−0.0240 (17)	0.0367 (18)	−0.0091 (17)
C15	0.0399 (17)	0.083 (2)	0.050 (2)	−0.0146 (17)	0.0292 (16)	−0.0107 (17)
C16	0.0357 (15)	0.0623 (19)	0.0350 (17)	−0.0046 (14)	0.0226 (14)	−0.0050 (14)
C17	0.0355 (14)	0.0444 (16)	0.0291 (15)	−0.0008 (13)	0.0203 (13)	−0.0025 (12)
C18	0.0373 (15)	0.0391 (15)	0.0315 (15)	0.0014 (12)	0.0224 (13)	−0.0012 (12)
C19	0.0457 (17)	0.0467 (17)	0.0358 (17)	0.0133 (14)	0.0271 (15)	0.0033 (13)
C20	0.0507 (19)	0.064 (2)	0.048 (2)	0.0203 (16)	0.0303 (17)	0.0051 (16)
C21	0.0359 (16)	0.079 (2)	0.0451 (19)	0.0125 (16)	0.0234 (15)	0.0017 (16)
C22	0.065 (2)	0.0359 (16)	0.0473 (19)	0.0130 (15)	0.0358 (17)	0.0019 (13)
C23	0.0609 (19)	0.0329 (16)	0.0479 (19)	0.0004 (14)	0.0321 (17)	−0.0024 (13)
C24	0.0462 (16)	0.0359 (16)	0.0477 (19)	−0.0007 (13)	0.0282 (15)	−0.0019 (13)
C25	0.0289 (14)	0.0428 (16)	0.0396 (17)	−0.0007 (12)	0.0201 (13)	−0.0018 (14)
C26	0.0284 (13)	0.0377 (15)	0.0392 (16)	0.0025 (12)	0.0196 (13)	0.0011 (12)
C27	0.0313 (14)	0.0457 (18)	0.0432 (18)	0.0026 (12)	0.0195 (14)	−0.0016 (13)
C28	0.0468 (17)	0.063 (2)	0.0461 (19)	−0.0013 (16)	0.0263 (16)	−0.0140 (16)
C29	0.065 (2)	0.076 (2)	0.045 (2)	−0.0119 (18)	0.0356 (18)	−0.0035 (18)
C30	0.085 (2)	0.057 (2)	0.050 (2)	−0.0113 (18)	0.042 (2)	0.0047 (17)
C31	0.062 (2)	0.0495 (19)	0.0443 (19)	−0.0095 (15)	0.0333 (17)	−0.0050 (15)
C32	0.0373 (15)	0.0504 (19)	0.0384 (17)	0.0010 (14)	0.0227 (14)	−0.0002 (13)
C33	0.0311 (14)	0.0488 (17)	0.0330 (16)	0.0065 (12)	0.0180 (13)	0.0033 (12)
C34	0.0482 (18)	0.054 (2)	0.0454 (19)	0.0092 (15)	0.0258 (16)	0.0042 (15)
C35	0.0411 (18)	0.080 (2)	0.054 (2)	0.0259 (17)	0.0260 (16)	0.0097 (17)
C36	0.0330 (16)	0.097 (3)	0.0443 (19)	0.0055 (17)	0.0231 (16)	0.0017 (17)
C37	0.0391 (17)	0.075 (2)	0.0426 (19)	−0.0039 (16)	0.0251 (15)	0.0011 (15)
C38	0.0367 (15)	0.0589 (19)	0.0315 (16)	0.0004 (14)	0.0203 (14)	0.0005 (13)

### Geometric parameters (Å, °)

**Table:**

Gd1—O6	2.4764 (17)	C8—C10	1.389 (4)
Gd1—O2	2.4781 (17)	C8—C9	1.409 (4)
Gd1—O5	2.4854 (16)	C10—C11	1.360 (4)
Gd1—O9	2.5039 (17)	C10—H10	0.9300
Gd1—N4	2.5433 (19)	C11—C12	1.384 (4)
Gd1—O1	2.5673 (16)	C11—H11	0.9300
Gd1—N3	2.5728 (19)	C12—H12	0.9300
Gd1—N1	2.5943 (19)	C13—C14	1.392 (4)
Gd1—O10	2.6022 (17)	C13—H13	0.9300
Gd1—N2	2.625 (2)	C14—C15	1.359 (4)
Gd1—C32	2.847 (3)	C14—H14	0.9300
Gd1—C25	2.910 (3)	C15—C16	1.401 (4)
O1—C25	1.270 (3)	C15—H15	0.9300
O2—C25	1.271 (3)	C16—C17	1.407 (3)
O3—C27	1.347 (3)	C16—C21	1.430 (4)
O3—H3	0.8201	C17—C18	1.438 (3)
O4—C31	1.355 (3)	C18—C19	1.402 (3)
O4—H4	0.8201	C19—C22	1.399 (4)
O5—C32	1.268 (3)	C19—C20	1.428 (4)
O6—C32	1.275 (3)	C20—C21	1.342 (4)
O7—C38	1.343 (3)	C20—H20	0.9300
O7—H7	0.8199	C21—H21	0.9300
O8—C34	1.361 (3)	C22—C23	1.348 (4)
O8—H8	0.8201	C22—H22	0.9300
O9—N5	1.275 (3)	C23—C24	1.394 (4)
O10—N5	1.252 (3)	C23—H23	0.9300
O11—N5	1.220 (3)	C24—H24	0.9300
N1—C1	1.336 (3)	C25—C26	1.473 (3)
N1—C5	1.353 (3)	C26—C31	1.406 (3)
N2—C12	1.321 (3)	C26—C27	1.414 (3)
N2—C9	1.360 (3)	C27—C28	1.380 (4)
N3—C13	1.331 (3)	C28—C29	1.368 (4)
N3—C17	1.363 (3)	C28—H28	0.9300
N4—C24	1.331 (3)	C29—C30	1.365 (4)
N4—C18	1.364 (3)	C29—H29	0.9300
C1—C2	1.402 (3)	C30—C31	1.384 (4)
C1—H1	0.9300	C30—H30	0.9300
C2—C3	1.349 (4)	C32—C33	1.480 (3)
C2—H2	0.9300	C33—C34	1.405 (4)
C3—C4	1.400 (4)	C33—C38	1.413 (3)
C3—H3A	0.9300	C34—C35	1.385 (4)
C4—C5	1.408 (3)	C35—C36	1.366 (4)
C4—C6	1.424 (4)	C35—H35	0.9300
C5—C9	1.442 (3)	C36—C37	1.379 (4)
C6—C7	1.338 (4)	C36—H36	0.9300
C6—H6	0.9300	C37—C38	1.390 (3)
C7—C8	1.436 (4)	C37—H37	0.9300
C7—H7A	0.9300		
			
O6—Gd1—O2	79.42 (6)	N1—C5—C9	118.2 (2)
O6—Gd1—O5	52.59 (6)	C4—C5—C9	119.0 (2)
O2—Gd1—O5	74.76 (6)	C7—C6—C4	121.4 (3)
O6—Gd1—O9	70.53 (6)	C7—C6—H6	119.3
O2—Gd1—O9	143.80 (6)	C4—C6—H6	119.3
O5—Gd1—O9	71.11 (6)	C6—C7—C8	121.4 (3)
O6—Gd1—N4	134.90 (6)	C6—C7—H7A	119.3
O2—Gd1—N4	72.04 (6)	C8—C7—H7A	119.3
O5—Gd1—N4	86.06 (6)	C10—C8—C9	118.0 (2)
O9—Gd1—N4	116.56 (6)	C10—C8—C7	123.3 (3)
O6—Gd1—O1	71.59 (6)	C9—C8—C7	118.7 (3)
O2—Gd1—O1	51.57 (5)	N2—C9—C8	122.0 (2)
O5—Gd1—O1	108.00 (6)	N2—C9—C5	118.1 (2)
O9—Gd1—O1	130.59 (6)	C8—C9—C5	119.9 (2)
N4—Gd1—O1	112.56 (6)	C11—C10—C8	119.8 (3)
O6—Gd1—N3	143.09 (6)	C11—C10—H10	120.1
O2—Gd1—N3	79.18 (6)	C8—C10—H10	120.1
O5—Gd1—N3	144.99 (6)	C10—C11—C12	118.5 (3)
O9—Gd1—N3	136.91 (6)	C10—C11—H11	120.7
N4—Gd1—N3	63.67 (6)	C12—C11—H11	120.7
O1—Gd1—N3	71.53 (6)	N2—C12—C11	124.4 (2)
O6—Gd1—N1	79.85 (6)	N2—C12—H12	117.8
O2—Gd1—N1	122.79 (6)	C11—C12—H12	117.8
O5—Gd1—N1	126.90 (6)	N3—C13—C14	123.1 (3)
O9—Gd1—N1	71.77 (6)	N3—C13—H13	118.5
N4—Gd1—N1	145.17 (7)	C14—C13—H13	118.5
O1—Gd1—N1	71.33 (6)	C15—C14—C13	119.3 (3)
N3—Gd1—N1	86.98 (6)	C15—C14—H14	120.3
O6—Gd1—O10	105.44 (6)	C13—C14—H14	120.3
O2—Gd1—O10	124.86 (6)	C14—C15—C16	120.3 (3)
O5—Gd1—O10	67.48 (6)	C14—C15—H15	119.8
O9—Gd1—O10	49.75 (6)	C16—C15—H15	119.8
N4—Gd1—O10	66.82 (6)	C15—C16—C17	116.6 (3)
O1—Gd1—O10	175.38 (6)	C15—C16—C21	124.0 (3)
N3—Gd1—O10	111.47 (6)	C17—C16—C21	119.4 (3)
N1—Gd1—O10	111.94 (6)	N3—C17—C16	123.3 (2)
O6—Gd1—N2	132.26 (6)	N3—C17—C18	117.6 (2)
O2—Gd1—N2	145.49 (6)	C16—C17—C18	119.0 (2)
O5—Gd1—N2	132.37 (6)	N4—C18—C19	122.5 (2)
O9—Gd1—N2	70.16 (6)	N4—C18—C17	117.4 (2)
N4—Gd1—N2	87.30 (6)	C19—C18—C17	120.2 (2)
O1—Gd1—N2	118.02 (6)	C22—C19—C18	117.6 (2)
N3—Gd1—N2	66.79 (6)	C22—C19—C20	123.8 (3)
N1—Gd1—N2	62.87 (7)	C18—C19—C20	118.6 (3)
O10—Gd1—N2	66.59 (6)	C21—C20—C19	121.7 (3)
O6—Gd1—C32	26.57 (6)	C21—C20—H20	119.1
O2—Gd1—C32	78.98 (6)	C19—C20—H20	119.1
O5—Gd1—C32	26.43 (6)	C20—C21—C16	120.8 (3)
O9—Gd1—C32	65.09 (7)	C20—C21—H21	119.6
N4—Gd1—C32	111.92 (7)	C16—C21—H21	119.6
O1—Gd1—C32	92.26 (7)	C23—C22—C19	120.0 (2)
N3—Gd1—C32	157.93 (7)	C23—C22—H22	120.0
N1—Gd1—C32	102.26 (7)	C19—C22—H22	120.0
O10—Gd1—C32	83.90 (7)	C22—C23—C24	119.0 (3)
N2—Gd1—C32	135.25 (7)	C22—C23—H23	120.5
O6—Gd1—C25	73.40 (6)	C24—C23—H23	120.5
O2—Gd1—C25	25.73 (6)	N4—C24—C23	123.6 (3)
O5—Gd1—C25	91.04 (6)	N4—C24—H24	118.2
O9—Gd1—C25	143.56 (7)	C23—C24—H24	118.2
N4—Gd1—C25	92.66 (7)	O1—C25—O2	119.6 (2)
O1—Gd1—C25	25.85 (6)	O1—C25—C26	120.8 (2)
N3—Gd1—C25	74.25 (6)	O2—C25—C26	119.5 (2)
N1—Gd1—C25	97.09 (7)	O1—C25—Gd1	61.84 (13)
O10—Gd1—C25	150.44 (7)	O2—C25—Gd1	57.80 (13)
N2—Gd1—C25	136.38 (6)	C26—C25—Gd1	176.71 (18)
C32—Gd1—C25	84.65 (7)	C31—C26—C27	117.2 (2)
C25—O1—Gd1	92.31 (15)	C31—C26—C25	121.4 (2)
C25—O2—Gd1	96.46 (15)	C27—C26—C25	121.4 (2)
C27—O3—H3	109.5	O3—C27—C28	117.7 (2)
C31—O4—H4	109.5	O3—C27—C26	121.5 (2)
C32—O5—Gd1	92.88 (15)	C28—C27—C26	120.8 (3)
C32—O6—Gd1	93.12 (15)	C29—C28—C27	119.5 (3)
C38—O7—H7	109.5	C29—C28—H28	120.2
C34—O8—H8	109.5	C27—C28—H28	120.2
N5—O9—Gd1	98.88 (13)	C30—C29—C28	121.9 (3)
N5—O10—Gd1	94.79 (14)	C30—C29—H29	119.0
C1—N1—C5	117.8 (2)	C28—C29—H29	119.0
C1—N1—Gd1	121.05 (18)	C29—C30—C31	119.3 (3)
C5—N1—Gd1	120.81 (15)	C29—C30—H30	120.3
C12—N2—C9	117.3 (2)	C31—C30—H30	120.3
C12—N2—Gd1	123.06 (17)	O4—C31—C30	117.9 (3)
C9—N2—Gd1	119.48 (16)	O4—C31—C26	121.0 (2)
C13—N3—C17	117.4 (2)	C30—C31—C26	121.1 (3)
C13—N3—Gd1	122.94 (17)	O5—C32—O6	119.6 (2)
C17—N3—Gd1	118.78 (15)	O5—C32—C33	120.5 (2)
C24—N4—C18	117.2 (2)	O6—C32—C33	119.9 (2)
C24—N4—Gd1	122.31 (16)	O5—C32—Gd1	60.70 (13)
C18—N4—Gd1	120.37 (15)	O6—C32—Gd1	60.30 (13)
O11—N5—O10	123.2 (3)	C33—C32—Gd1	166.12 (18)
O11—N5—O9	120.3 (2)	C34—C33—C38	118.3 (2)
O10—N5—O9	116.6 (2)	C34—C33—C32	121.3 (2)
O11—N5—Gd1	175.9 (2)	C38—C33—C32	120.4 (2)
O10—N5—Gd1	60.47 (12)	O8—C34—C35	117.9 (3)
O9—N5—Gd1	56.11 (11)	O8—C34—C33	120.9 (2)
N1—C1—C2	122.6 (3)	C35—C34—C33	121.1 (3)
N1—C1—H1	118.7	C36—C35—C34	118.5 (3)
C2—C1—H1	118.7	C36—C35—H35	120.7
C3—C2—C1	119.6 (3)	C34—C35—H35	120.7
C3—C2—H2	120.2	C35—C36—C37	123.1 (3)
C1—C2—H2	120.2	C35—C36—H36	118.5
C2—C3—C4	119.9 (3)	C37—C36—H36	118.5
C2—C3—H3A	120.0	C36—C37—C38	118.7 (3)
C4—C3—H3A	120.0	C36—C37—H37	120.7
C3—C4—C5	117.4 (3)	C38—C37—H37	120.7
C3—C4—C6	123.0 (3)	O7—C38—C37	117.1 (2)
C5—C4—C6	119.6 (3)	O7—C38—C33	122.6 (2)
N1—C5—C4	122.8 (2)	C37—C38—C33	120.3 (3)

### Hydrogen-bond geometry (Å, °)

**Table:**

*D*—H···*A*	*D*—H	H···*A*	*D*···*A*	*D*—H···*A*
O7—H7···O5	0.82	1.87	2.592 (2)	147
O8—H8···O6	0.82	1.83	2.563 (3)	148
O4—H4···O1	0.82	1.86	2.585 (3)	147
O3—H3···O2	0.82	1.84	2.574 (3)	148
